# Virtual Reality–Based Food and Beverage Marketing: Potential Implications for Young People of Color, Knowledge Gaps, and Future Research Directions

**DOI:** 10.2196/62807

**Published:** 2024-10-17

**Authors:** Omni Cassidy, Marie Bragg, Brian Elbel

**Affiliations:** 1 Department of Population Health, NYU Grossman School of Medicine, 3rd Floor, 180 Madison Avenue, New York, NY, 10016, United States, 1 6465013546; 2 Marketing Department, NYU Stern School of Business, New York, NY, United States; 3 NYU Wagner Graduate School of Public Service, New York, NY, United States

**Keywords:** virtual reality, VR, digital food and beverage marketing, obesity, marketing, food, consumption, beverage, immersive, market, consumer

## Abstract

Exposure to unhealthy food and beverage marketing is a major contributor to excessive weight gain among young people and it may disproportionately affect Black and Latinx communities. Appropriate and comprehensive regulations on food and beverage companies are essential, particularly as companies expand their reach and leverage the latest technologies to create marketing experiences using immersive virtual reality (VR). Although immersive VR technology is in its infancy, the potential effects of immersive VR food and beverage marketing on consumption, coupled with the history of racially targeted marketing by food and beverage corporations toward Black and Latinx communities, and the heightened burden of diet-related illnesses in Black and Latinx communities underscore a critical need to investigate immersive VR marketing targeting young people of color. This viewpoint will provide a brief description of VR food and beverage marketing as the newest food and beverage marketing frontier, highlight key concerns and knowledge gaps, and underscore future directions in research.

## Introduction

Exposure to unhealthy food and beverage marketing is a major contributor to excessive weight gain in youth [1,2]. Youth exposed to food and beverage advertising (“ads”) demonstrate more preferences for advertised foods, higher purchase intention, and increased food consumption compared to youth exposed to nonfood ads [3-9]. Data examining food marketing on digital platforms (eg, social media and gaming) also show influences on food preferences and consumption [10-17]. Although most food marketing studies focus on children due to their inability to recognize persuasive intent [5], adolescents (12‐17 years old) [18,19], and young adults (18‐34 years old) may also be highly targeted in this new digital era [20,21]. A few countries have made some progress in enacting restrictions on youth-targeted food marketing, with the majority of protections going up to only 16 years of age [22]. Many older adolescents and young adults of color in the United States remain unprotected from the full spectrum of unhealthy food and beverage marketing, placing them at increased risk for poor diet, excessive weight gain, and diet-related illnesses [22-25]. The failure to adopt comprehensive protections is particularly concerning as companies expand their reach and leverage the latest technologies to create new, more powerful marketing experiences using virtual reality (VR) [26,27]. Although VR technology is in its infancy, the potential effects of VR food and beverage marketing on consumption [28], food and beverage corporations’ history of racially targeted marketing to Black and Latinx communities in the United States [26,29], and heightened burden of diet-related illnesses in Black or Latinx communities underscores a critical need to investigate VR marketing to adolescents and young adults of color. This viewpoint will provide a brief description of VR food and beverage marketing as the newest food and beverage marketing frontier, highlight key concerns and knowledge gaps, and underscore future directions in research. Although unhealthy food and beverage marketing has wide implications for populations of color globally [30-32], this viewpoint will focus primarily on US populations.

## The New Digital Food and Beverage Marketing Landscape

Over the last 20 years, there has been a dramatic shift in the food and beverage marketing landscape. Food and beverage marketing expenditures toward television are declining while digital platforms are increasing [28,33,34]. Although reports indicate that food and beverage companies continue to allocate the majority of their marketing expenditures to television [34], expenditure reports may mask actual exposure in the current digital environment. In 1 study where adolescents (13‐17 years old) in Canada were asked to capture and report all forms of food and beverage marketing exposure over a 7-day period, over 90% (1681/1825 ads) of exposure occurred on digital platforms (eg, social media) versus under 2% (31/1825 ads) on traditional television [35]. The lower costs of digital marketing relative to traditional television marketing have allowed companies to expand their reach while minimizing costs [33].

The early stages of this transformation were noted in a 2009 review by Montgomery et al [36]. Authors indicated how food and beverage companies were beginning to leverage adolescents’ engagement with social media, gaming, and immersive or virtual words [36]. Growing concerns prompted agencies, such as the Federal Trade Commission (FTC) in the United States, to investigate the role of television versus digital food and beverage marketing to youth [27,37]. In 2012, the FTC reported that between 2006 and 2009, expenditures toward youth-targeted television food and beverage marketing (defined as marketing directed at individuals 2‐17 years old) declined by 20%, while online food and beverage marketing expenditures increased by 50% [38]. Other leading health organizations, such as the World Health Organization [39], also began recognizing that food and beverage companies were using new digital strategies (eg, websites). Yet, research and policy continued to focus primarily on television exposure.

Due in large part to food and beverage corporations’ expansive financial resources and political involvement [40], food and beverage companies and marketers’ use of sophisticated digital marketing practices grew rapidly while research monitoring digital food and beverage marketing grew slowly [17]. Marketing strategies evolved into “AdTech” that combined neuromarketing and consumer data to create automated, personalized, and contextual ads powered by artificial intelligence or machine learning and algorithmic programming tools [26,33,41]—advancements that were propelled by the COVID-19 pandemic. Social media food and beverage marketing increased as did promotional content on gaming and streaming platforms [16,26,34,41,42]. The first food and beverage companies began posting on social media in 2007, and between 2007 and 2016, account creation increased by 567% (n=1 in 2007 to n=568 in 2016) [43]. As part of their social media campaigns, companies also began using “influencers,” everyday people taking on celebrity status on social media platforms [16,26,34,41,42].

Now 10 years after the predictions from consumer agencies that food and beverage companies would begin leveraging technological advancements [28,33,34,38], digital food and beverage marketing has grown into a “wicked problem”—highly complex, difficult to identify and understand, and where effective solutions to reduce or eliminate its negative impact are not straightforward [33,44]. Digital food and beverage marketing has infiltrated every digital and streaming-based platform while also maintaining a “black box” on data gathered, making it difficult to both determine the actual exposure among young people with specificity and digital marketing’s influence on purchasing [33]. The current landscape begs the question—what will the next 10 years hold if unhealthy food and beverage marketing continues to expand and evolve in underregulated ways on digital platforms? This concern is particularly relevant when coupled with concerns about food and beverage companies’ continued efforts to target Black and Latinx consumers [26,37].

## Corporations’ History of Racially Targeted Marketing to Black and Latinx Consumers in the United States

Racially targeted marketing refers to practices that disproportionately advertise unhealthy foods and beverages to Black or Latinx consumers, and is often rooted in structural racism and discrimination within business models [29,45]. Racially targeted marketing typically involves placing ads on Black or Latinx-oriented television programs [46,47] or embedding ads with culturally salient cues that may appeal to Black and Latinx communities, such as racially congruent actors, activities with a high percentage of Black or Latinx participants (eg, basketball and Spanish heritage holidays) [48-50], or popular Black or Latinx musicians or celebrities. Companies also increase the appeal of ads targeting Black and Latinx consumers by exploiting price sensitivity due to income inequities, and the communities’ greater trust and responsiveness to marketing after a history of being excluded from the marketplace and advertising [29,34]. Food and beverage companies have a history of disproportionately targeting Black and Latinx consumers in the United States with unhealthy food and beverage marketing [34,51], contributing to excessive weight gain and diet-related health disparities [25]. Black youth (2‐17 years old) are exposed to 2 times more unhealthy food ads compared to White youth [34]. Ads targeting Black and Latinx consumers tend to be less healthy compared to ads targeting other audiences [29,46,47]. Ads in predominantly Black and Latinx neighborhoods [52], or that feature Black or Latinx actors, often promote fewer healthy foods compared to ads targeting non-Black or Latinx audiences [29,46,47].

Research on how food and beverage companies target Black and Latinx communities on digital platforms is growing [19,34,53-57]. Advocacy reports suggest that companies are becoming more sophisticated in their targeting approaches [26,27,37]. “Multicultural” AdTech campaigns, as they are termed, are being developed to target Black and Latinx adolescents as the next generation of savvy tech users [26]. These digital ads can follow adolescents across their devices and deliver personalized content based on geolocation, purchasing patterns, and the racial or ethnic mix of their neighborhood [26]. Bilingual Latinx youth are sometimes targeted as the translators (referred to as “sherpas”) of new technologies and brands for their families [26]. These efforts, coupled with the access to consumer data and technologies that allow for sophisticated analyses, underscore the need to address digital marketing among Black and Latinx communities. Although we are beginning to uncover the techniques and effects of social media marketing [10,55-57], strategies within immersive or virtual environments are completely unchartered.

## VR Food and Beverage Marketing: The Newest Marketing Frontier

Against this historical backdrop, this viewpoint centers on 1 evolution within digital marketing, immersive marketing using VR technology. VR uses AI or machine learning to create a computer-simulated environment where individuals are psychologically transported and submerged into a life-like world (immersion). Levels of immersion are dependent on the degree to which a VR system delivers an experience that (1) removes cues from physical reality (inclusive), (2) provides a range of sensory integration (extensive), (3) engulfs one’s field of view (surrounding), and (4) provides rich and high-quality content (vivid) [58-60]. VR equipment can provide immersive experiences that are low-immersive (eg, computer desktop), semi-immersive (eg, video wall that includes images projected onto a large screen), or full (eg, head-mounted displays that engulf the field of view and present continually updated visual stimuli based on the users’ movements and selections) [58]. Based on the immersion level, VR experiences are designed to engender varying senses of “being” in the digital world (presence) and feeling as though one exists in the digital body (embodiment) [61]. Immersion, presence, and embodiment are core features that make VR fundamentally different than other forms of media, such as television or radio [62].

Based on the food and beverage cues in the digital marketing model [28], exposure to more immersive VR food and beverage marketing may have unique effects on consumers’ perception through 3 pathways. First, increasing saturation, allows multiple elements to be present on a young person’s screen simultaneously and embedded in the content being viewed. Second, increasing the level of synergy and congruency between marketing and entertainment which continuously blurs the lines between marketing and entertainment. Third, capitalizing on the strong social engagement present in most digital media platforms [28]. VR food and beverage marketing, therefore, is theorized to create lasting effects on consumption and health [28]. Scientific data on current consumer engagement with fully immersive VR are lacking. Available data, however, demonstrate that popularity appears to be growing. According to market reports, as of 2024, approximately 25% (over 66 million) of US adults reported ever or currently using fully immersive VR (via head-mounted display), up from 16% in 2019 [63,64]. Two-thirds of fully immersive VR users are 16‐34 years old, making adolescents and young adults the dominant users of fully immersive VR (34% are 16‐23 year olds, 35% are 24‐34 year olds) [65]. Like other emerging technologies, uptake in the general population has not occurred linearly. Initially developed in the 1960s, fully immersive VR technology has seen more growth in the last 2 years than the previous decade, driven by the introduction of low-cost hardware, technology advancements that create more life-like experiences [66], global digitization precipitated by the COVID-19 pandemic, and investments into immersive experiences by companies like Apple [67].

Food and beverage companies have joined other industries in developing VR marketing experiences at varying immersion levels that are engaging and highly sophisticated [26,27,37,41,68]. Food companies’ early iterations of VR experiences were on gaming platforms that incorporated VR capabilities. Wendy’s—one of the top fast food restaurants advertising to adolescents [69]—created a game that was only available 1 time for 9 hours within the popular videogame Fortnite (termed “limited time mode”). The game—which could be played at a wide range of immersion levels—featured a main character whose mission was to destroy all freezers and was built around Wendy’s slogan, “fresh, never frozen, beef” [70]. In the 9 hours the limited time mode game was live, it was viewed for 1.5 million minutes; streamed 250,000 times; and garnered 23 million impressions (the total number of times the content has been shown to users) [26].

As of April 2024, Chipotle [71,72], Wendy’s [73,74], McDonald’s [75], and Taco Bell [76] have published VR experiences with apps designed for full immersion via head-mounted displays, although they can also be accessed at lower immersion levels via a computer desktop (Textbox 1). It has been reported that Burger King, KFC, Pizza Hut, Chick-fil-A, Hooters, Dunkin Donuts, and Panera have filed trademark applications to create VR and related experiences [77]. As described in Textbox 1, some experiences focus explicitly on brand engagement (Wendy’s and Chipotle), while others promote cultural events (Taco Bell and McDonald’s). In 2022, Wendy’s became the first to develop a stand-alone branded VR world, the “Wendyverse,” which was reported to be the most visited virtual world on the Meta platform and garnered over 650 million impressions [73,74]. Food and beverage VR experiences appear to be a growing phenomenon that may represent the latest evolution in digital food and beverage marketing [75].

Textbox 1. **Brief description of virtual reality marketing experiences developed by Wendy’s, Chipotle, McDonald’s, and Taco Bell from 2019 to 2023.**

- Wendy’s (2019): limited time mode game, “Food Fight,” within the popular videogame, Fortnite, with a character whose mission was to destroy all freezers. The campaign was built around their slogan, “fresh, never frozen, beef.”
- Chipotle (2021): promotional virtual reality (VR) experience, the “Chipotle Boorito Maze,” for users of the online gaming platform, Roblox, a 3D gaming environment popular among young adults. Users could follow a maze and receive daily in-game codes to purchase burritos at a Chipotle in the real world.
- Chipotle (2022): a promotional VR experience in 2022 on Roblox, the “Chipotle Burrito Builder,” where users could build their own burrito with a virtual chef.
- Wendy’s (2022): first stand-alone branded VR world, the “Wendyverse,” which is already the most visited virtual world on the Meta platform and garnered over 650 million impressions. Users are transported to a virtual world where they can visit a restaurant that mimics those in the real world, play basketball with a Wendy’s bacon cheeseburger instead of a basketball, socialize with others at a town square, and access give-a-ways they can use at restaurants in the real world.
- McDonald’s (2022) : McDonald’s created a virtual Lunar New Year Celebration where visitors could view statues of the 12 zodiac signs and have their horoscopes read.
- Taco Bell (2023): Taco Bell created a contest for engaged couples to apply to have their wedding in a Taco Bell virtual world within the metaverse. The couple who reportedly shared a “passion for Taco Bell” were married in the metaverse on February 22, 2023.

## Knowledge Gaps

There is a paucity of data on the extent to which companies are targeting adolescents and young adults of color with fully immersive VR food and beverage marketing. Recent reports suggest that 20% of Latinx and 11% of Black consumers are engaging with fully immersive VR using head-mounted displays (compared to 61% of White consumers) [78]. Notably, there may be barriers to uptake among Latinx and Black consumers, such as cost. This barrier may affect the perceived ease of use—one of the key components necessary to accept VR as a new technology, according to the technology acceptance model [79]. Even though VR technology may have limited uptake in vulnerable populations today, there are several reasons it is important to focus on this growing phenomenon. First, food or beverage companies have a history of targeting young people and young people of color, which has evolved and likely intensified with technological advancements, such as geolocation [29,68]. Second, while adolescents and young adults of color may not be the earliest adopters of VR technologies, young people are highly engaged in digital media and Black consumers, in particular, tend to have higher engagement with media compared to the general population [80]. Third, focusing on this issue now allows professionals to anticipate health needs in vulnerable communities rather than react to these needs as they arise. Technological advancements move at a rapid speed. For instance, in 1983, less than 2% of the US adults used a home computer. Seven years later, reported usage increased to 42% [81]. By 2018, 92% of US households had a home computer. Finally, it takes time to develop, conduct, and complete sufficient research to inform policies that also take many years to materialize. If we wait until VR marketing has already taken root within these communities, the harms may be harder to address.

## Future Research Directions and Policy Considerations

More research is needed to understand the effects of exposure to VR food and beverage marketing and marketing at varying levels of immersion on young people of color. The research needed includesbut is not limited to, effects on preferences, purchase intention or “pester power” among younger children, purchases, and consumption [3-9]. Content analyses on VR marketing experiences are necessary to capture the unique elements that may appeal to young people of color. Additionally, given the possibility that VR marketing may bypass conscious awareness at higher levels of immersion [26], it is critical to conduct research examining the effects on subconscious processes, including brain function, heart rate, facial expression, and arousal. Leveraging objective methods such as eye-tracking, electroencephalography, functional magnetic resonance imaging, facial expression recognition software, functional near-infrared spectroscopy, electrocardiogram, and electrodermal activity or skin conductance hold promise [82,83]. It may also be important to understand the comparative and cumulative effects of marketing from VR, social media, gaming, and television. Finally, as unhealthy food and beverage marketing in virtual environments becomes more of a reality, effective counter-marketing strategies may need to work in tandem with comprehensive policies that support communities of color in navigating these new environments. Filling these research gaps can inform the development of comprehensive policies that address the full spectrum of digital food and beverage marketing to adolescents and young adults of color [22].

## Conclusions

Despite growing concerns worldwide [22], the expansion of food and beverage companies into the digital landscape has created a “wicked problem” with few straightforward solutions. Given food and beverage companies’ history of targeted marketing, adolescents and young adults of color may be particularly vulnerable to the ways companies have used technological advancements to develop VR food marketing experiences. More research is needed to monitor adolescents and young adults of color’s exposure to fully immersive VR food marketing and the unique effects of fully immersive VR food marketing on physiological (eg, arousal), cognitive (eg, attention), and behavioral (eg, consumption) outcomes. This early stage of VR development provides a valuable opportunity to begin filling these research gaps and inform comprehensive policies that can support a better food marketing environment for younger generations over the next 10 years.

## Abbreviations

FTC: Federal Trade Commission
VR: virtual reality
